# *PiezoGRIN*: A High‐Pressure Chamber Incorporating GRIN Lenses for High‐Resolution 3D‐Microscopy of living Cells and Tissues

**DOI:** 10.1002/advs.201801453

**Published:** 2018-12-14

**Authors:** Dominik Schneidereit, Sebastian Schürmann, Oliver Friedrich

**Affiliations:** ^1^ Institute of Medical Biotechnology Friedrich‐Alexander University Erlangen‐Nürnberg Paul‐Gordan Strasse 3 Erlangen 91052 Germany; ^2^ Erlangen Graduate School in Advanced Optical Technologies (SAOT) Friedrich‐Alexander‐University Erlangen‐Nürnberg Erlangen 91052 Germany; ^3^ Muscle Research Center Erlangen (MURCE) Paul‐Gordan Strasse 3 Erlangen 91052 Germany

**Keywords:** high pressure, multiphoton microscopy, second harmonic generation, skeletal muscle

## Abstract

A high‐pressure optical chamber, *PiezoGRIN*, that facilitates label‐free 3D high‐resolution live‐cell multiphoton microscopy in thick tissue samples is presented. A set of two Gradient Index (GRIN) rod lenses is integrated into the chamber as an optical guide and allows for the adjustment of the focal plane through the sample providing a field of view volume of 450 × 450 × 500 µm (*x*, *y*, *z*). An optical lateral resolution of 0.8 µm is achieved by using two‐photon excitation with 150 fs pulses of a 810 nm titanium–sapphire laser at hydrostatic pressures up to 200 MPa. With the *PiezoGRIN* setup, it is possible to follow pressure‐induced changes in subcellular structure of unstained vital mouse skeletal muscle tissue up to 200 µm below the tissue surface.

## 1 Introduction

In biomedical research, unlike temperature, the environmental variable of pressure is often neglected and processes that are observed at atmospheric pressure are considered to represent a standard for which the temperature dependence can be assessed. Assuming 1 atm (0.1 MPa) as the universal standard pressure for all biological organisms may be misleading as less than 1% of Earth's biosphere volume is exposed to atmospheric pressure or below, whereas 79% is exposed to at least 10 MPa of hydrostatic pressure.^1^ Earth's surface is covered to about 70 % with ocean at an average depth of 3700 m, resulting in an average hydrostatic pressure of 37 MPa.^2^ A major reason for neglecting pressure in biosciences is that establishing high‐pressure environments experimentally is usually neither simple nor cheap. In previous observations of high‐pressure effects on isolated mouse muscle cells,^3^ the need for an improved optical high‐pressure vessel, allowing for the analysis of subcellular morphology^4^ in whole tissue emerged. To that end, we present the optical high‐pressure vessel *PiezoGRIN*, engineered from inexpensive materials, which allows label‐free 3D high‐resolution multiphoton microscopy at elevated hydrostatic pressure.

## 2 Results

**Figure 1:**
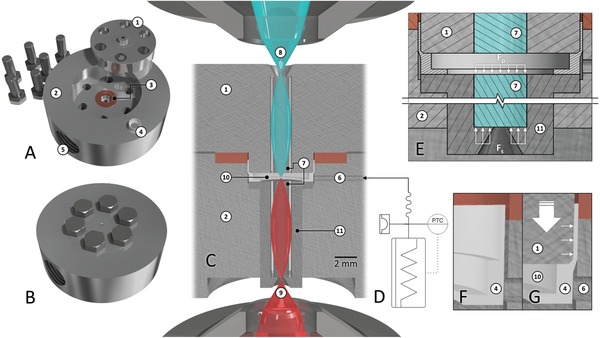
Detailed illustration of the *PiezoGRIN* chamber and its periphery. A) View of the *PiezoGRIN* system with the lid (1) disassembled from the chamber body (2) and removed PTFE ring (4) that is used to separate the sample volume from hydraulic fluid. A copper gasket (3) is used as‐high‐pressure seal, the chamber is connected to the pressure generator via a standard 16 mm fitting with a barrel gasket seal (5). B) Closed chamber assembly with 6 M6 Screws tightened using 8 Nm torque. C) Half‐cut view of assembled *PiezoGRIN* chamber showing excitation light (9) focused by an objective, entering the chamber through a GRIN rod lens (7) and emitted signal (8) exiting the sample volume (10) through a second rod lens and being collected by a second objective. The excitation GRIN lenses are mounted in an exchangeable bolt (11) to allow for easy replacement. A pressure capillary (6) transmits hydrostatic pressure, applied by the pressure generator, by means of hydraulic fluid onto the PTFE gasket surrounding the sample volume. D) Flowchart of pressure generator showing spindle press, pressure sensor and flexible high‐pressure hose. E) Schematic half‐cut view of excitation side GRIN lens (7), illustrating how pressure force *F*_p_ contributes to generate a Poulter‐type high‐pressure seal with sealing force *F*_s_. F) Illustration of the PTFE sealing ring (4) before and G) after initial setting. The ring is deformed by the chamber lid (1) and forms a low‐pressure seal (Δ*P* 0.1 MPa), separating pressure capillary (6) and sample volume (10).

In conjunction with the applied multiphoton microscopy setup, the chamber provides a lateral optical resolution of 0.8 µm in the focal plane and 7.4 µm axial resolution using two‐photon excitation with approximately 0.2 nJ femptosecond pulses at 810 nm at hydrostatic pressures up to 200 MPa. The resolution is defined as the full‐width at half maximum (FWHM) of the point‐spread function (PSF). Instead of the usually applied sapphire^5^ or diamond^6^, ^7^ windows, the chamber contains a set of two GRIN rod lenses, integrated into the chamber body and lid (**Figure** **1**C). The removable lid forms a high‐pressure flat seal with a copper gasket when assembled (Figure 1 A,B). Pressure is generated by a spindle press, conducted via hydraulic fluid through a flexible high‐pressure hose and measured by a piezoelectric sensor near the generator as shown in the flowchart of Figure 1D. A two‐barrier solution assures separation of sample and hydraulic fluid. The separation concept entails a polytetrafluoroethylene (PTFE) ring inset in the sample chamber as first barrier (Figure 1F,G) and a petroleum jelly clot in the pressure capillary as a second barrier.

The employed cylindrical GRIN lenses feature a radial distribution of refractive index that follows a hyperbolic secant (SECH) function while maintaining a constant refractive index in axial direction. Each lens pair projects an intermediate image of the sample to the outside, thus effectively granting additional working distance to any microscope objective used for imaging. Additionally, the lenses allow for 10 mm wide chamber walls to withstand the pressure by creating an optical access with a high length‐to‐diameter ratio of 5 while still retaining a comparably large field of view (FOV) of 450 µm and numerical aperture (NA) of 0.5. A roughly 30 µm thick layer of cured optical glue fixes the lenses and provides a low‐pressure seal. Similar to the glass disks in the design of Nishiyama^8^ the GRIN lenses are forming a Poulter‐type seal in their seating, using the pressure force as sealing force and the optical glue as gasket (Figure 1E).

**Figure 2:**
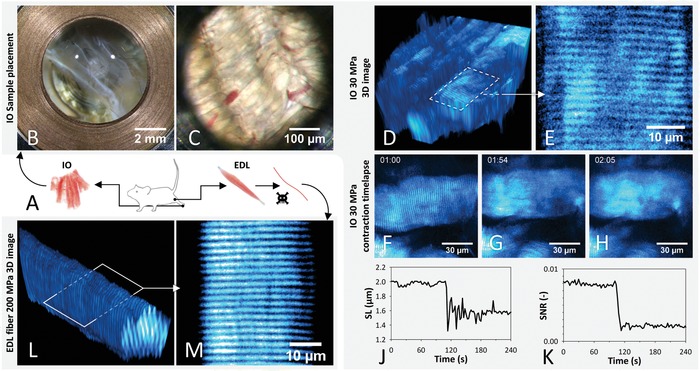
*PiezoGRIN* in action, showing muscle single fiber and whole tissue morphology at hydrostatic pressure. A) Illustration of the extraction of *murine* IO and EDL as well as fixing of EDL single fibers. B) Immersed IO tissue in open chamber with background light coming through the lens in the center and discernible copper seal in the image perimeter. C) Bright‐field image of IO tissue in closed chamber showing muscle fibers and blood vessels in an FOV of 0.5 mm. D) 3D image stack (115 × 115 × 43 µm) of unstained live IO tissue at 30 MPa, obtained by recording transmitted SHG signal of 2P excitation. E) Cropped slice from within stack (D) is showing SHG signal of A‐bands of the intracellular sarcomere structures from myofibrils. F–H) Time‐lapse (mm:ss) SHG images of live IO in calcium free HKS solution contracting after compression to 30 MPa. The compression phase concludes at *t* = 1 min, contracture of the fiber occurs just before the 2 min mark. J) The course of the average SL and K) Average SNR in the recorded images show a clear drop of both parameters just before the 2 min mark. L) 3D stack (115 × 115 × 101 µm) generated by 2P excitation of unstained fixed murine EDL single fiber and recording SHG signal in transmission direction at a hydrostatic pressure of 200 MPa. M) An enlarged central slice of the recorded stack shows the striation pattern of A‐band SHG signal and makes a good example of the high‐pressure high‐resolution 2P‐Imaging capabilities of our setup.

As proof of concept, fixed single mouse *extensor digitorum longus* (EDL) muscle single fibers and whole intact *musculi interossei* (IO) (**Figure** **2**A) tissue are imaged using label free 3D multiphoton microscopy at pressures up to 200 MPa. Fixed single EDL fibers show no pressure‐induced change in cellular morphology, and even at 200 MPa (Figure 2L,M), the cellular actin–myosin architecture^9^ is clearly revealed by 2P excitation. The 3D structure of live IO tissue is imaged in calcium free high K^+^ solution (HKS) (Figure 2D,F) and previously reported contracture at pressure near 30 MPa due to pressure‐induced deregulation of the troponin C regulatory protein complex of the contractile apparatus in conjunction with putative pressure‐induced SR Ca^2+^‐leak^3^, ^10^ is observed in detail, revealing an onset of fiber contracture after ≈1 min of exposure to 30 MPa in the time‐lapse (mm:ss) Figure 2F,G. During the contracture, the mean sarcomere length (SL) of the observed fibers decreases by 0.4 µm from 2.0 ± 0.03 to 1.6 ± 0.11 µm, and the signal‐to‐noise ratio (SNR) of the sarcomere second harmonic generation (SHG) signal decreases by 62% of its initial value, which is shown in the line graphs of Figure 2J,K. The time until contracture varies between individual fibers, as may be seen in the Video S1 in the Supporting Information. There, the contracture of the fiber in the center of the FOV is followed by several contractures of surrounding fibers. This suggests differential susceptibility to pressure‐induced contracture initiation in individual fibers in the intact muscle.

## 3 Discussion

**Figure 3:**
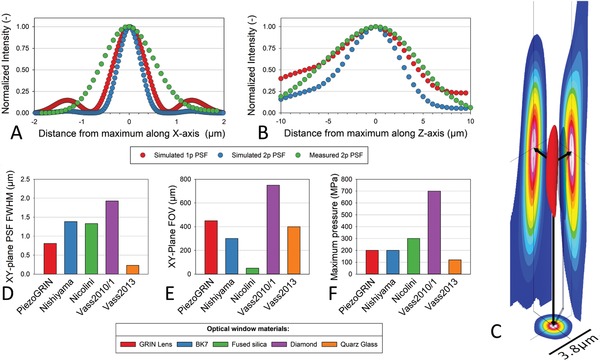
PSF of *PiezoGRIN* and comparison of its parameters to similar published high‐pressure vessels by Nishiyama,^8^ Nicolini et al.,^11^ and Vass et al.^7^, ^12^ Parameters of other systems that are not specifically detailed in the publications are estimated based on provided data. A) Scatter plot of PSFs along the *X*‐axis that represent sequential ray tracing simulations of 1p and 2p on‐axis excitation in the *PiezoGRIN* setup and a measured PSF using 2p excitation. B) On axis scatter plots of 1p and 2p excitation PSFs along Z‐axis of the *PiezoGRIN* system, generated by sequential ray tracing, show an asymmetric peak that is fronting towards the excitation direction. The fronting effect is well reproduced by the recorded 2p PSF. C) A 3D representation of a measured example PSF in the *PiezoGRIN* system is shown in red with projections of each plane‐PSF in a false color gradient. D) Bar graph comparing the FWHM of the PSF in the *XY*‐plane of *PiezoGRIN* to other systems, showing that it offers a higher than average *XY*‐resolution with a mean PSF of 0.54 µm E) The bar graph shows that the *PiezoGRIN* provides an FOV in the XY‐plane that is above the average of other comparable systems due to the integrated refractive elements. F) When compared to other systems *PiezoGRIN* shows a lower than average maximum pressure range due to the lens glass material with relatively low mechanical strength.

The presented *PiezoGRIN* system, in conjunction with the two‐photon excitation microscope, allows a detailed microscopic assessment of single cells and deep into vital whole tissue while applying hydrostatic pressure, without having to rely on altering the sample by staining procedures. In spite of the 10 mm thick chamber walls, the system features well‐confined PSFs perpendicularly (**Figure** **3**A) and longitudinally (Figure 3A) to the incident light. The PSF was determined by recording the emission of subresolution size fluorescent beads that provide a point light source and subsequent software analysis of recorded images, as elaborated in the Experimental Section. Even though the comparison of the measured system PSFs with on‐axis sequential ray tracing simulations shows that the optical performance is not perfect, the measurements coincide quite well with the simulations. The mean measured PSF FWHM with 0.8 µm is about 51% larger in the *XY*‐plane and with 7.4 µm about 11% larger along the *Z*‐axis than the simulated 2p PSF FWHMs. A reason for the discrepancies might be that the simulated PSFs are generated exactly on the optical axis and at optimal working distance from the GRIN lenses, while the measured beads were randomly distributed within the imaged 3D space of 780 µm^3^. PSF asymmetries, which become more apparent in lower intensity ranges and are visualized in Figure 3C, indicate additional aberrations that are possibly caused by slight misalignments in the optical system, promising a potential increase in optical performance by use of a single GRIN lens with a pitch 0.46 instead of the here applied lens pair with a pitch of 0.23 each.

The resulting perpendicular mean PSF, however, is still smaller than the PSFs of most other comparable systems^7^, ^8^, ^11^, ^12^ (Figure 3D). As an additional advantage, the image plane is not restricted to the interface between GRIN lens and sample fluid but may be moved up to 500 µm into the sample chamber. This allows for imaging through most of the height of the sample volume. Most similar systems do not provide sufficient data to estimate their optical performance along the *z*‐axis, with the exception of Vass et al.^7^ It is estimated that the *z*‐axis resolution of *PiezoGRIN* is about 20% higher than the previously published vessel, based on approximated PSF FWHMs.

Previous high‐pressure optical systems have used a variety of window materials that have been selected to provide high‐pressure stability while maintaining sufficient optical performance. In traditional window systems, there are severe limitations on window thickness due to the requirement of the sample being within the intrinsic working distance of the objective lens used. While CVD diamond offers a very high tensile strength of 750 MPa^13^ and its optical transmission is excellent from the UV to NIR range,^14^ birefringence values greater than 0.001 Δ*n* are not uncommon^15^, ^16^ in the material and may be distributed erratically while also being strain dependent.^17^, ^18^ While birefringence is usually a parameter of only secondary importance, it becomes more of an issue in high‐resolution microscopy, especially in the case of multiphoton excitation applications, as focal spot quality and confinement and thus, signal intensity and resolution may be reduced significantly.^19^, ^20^ This warrants a careful application of the CVD material in optical systems that require well defined focal spots. Keeping birefringence in mind, other mechanically strong, transparent materials such as sapphire and quartz with Δ*n* > 10^−3^ 21 are also poorly suited for high precision optics. To mitigate the birefringence problem, it appears favorable to utilize optical glasses such as BK7, as applied by Nishiyama^8^ which in turn however, requires a high window thickness with a small aperture due to a lower mechanical strength of the material than even fused silica.^22^ This results in either a reduced optical access angle to the sample, decreasing spatial resolution, or a small FOV at relatively low maximum pressure rates (see Figure 3F). Due to the isotropic character of borosilicate glass, it is not generating birefringence under standard conditions. However, strain‐induced birefringence is reported in BK7, but at about one order of magnitude less pronounced than in aforementioned materials.^23^ To reduce the hydrostatic pressure‐induced stress on the material, a cylindrical form factor with a high length‐to‐diameter ratio is advantageous. GRIN lenses that are usually fabricated from borosilicate glasses, feature that kind of form factor and offer the additional benefit of modifying the beam path preferably, so a relatively large FOV and optical access angle to the sample may be maintained despite of a layer thickness of the optical window in the range of 10 mm. Despite the fact that GRIN lenses generate additional low level birefringence, resulting from their production process^24^, ^25^ the advantages in optical and mechanical properties the lenses provide render them a suitable low‐cost alternative to conventional high‐pressure windows. Combining the *PiezoGRIN* with multiphoton excitation microscopy,^26^ we were able to visualize the effects of hydrostatic pressure on cellular substructures in vital intact muscle tissue samples in real time for the first time, featuring tissue penetration depths of up to 200 µm while not having to rely on staining or microsectioning procedures. By providing a full set of blueprints (S2) of the system, we are confident to be able to offer a reliable low‐cost platform for high‐pressure studies to lower the barrier to using pressure as an environmental variable in experiments on biological organisms or tissues.

## 4 Experimental Section

The chamber was manufactured from 1.4104 (X14CrMoS17) martensitic stainless steel that offered high offset yield strength (R_p0.2_ 500) and tensile strength (R_m_ 700) while maintaining good machinability and corrosive resistance at low cost. Design and finite element simulation of the chamber was performed in Autodesk Inventor 2016. A set of blueprints of the chamber was available in the supplemental file S2. A 12 × 6 × 1 mm DIN 7603A copper ring provided a high‐pressure seal when the six M6 DIN EN 24 017 screws were tightened with a torque of 8 Nm.

An automated single piston spindle press (Dustec Type 511.017.0160.040.TS) with 16 mL stroke volume was used for pressure generation and offered a pressure adjustment precision of ±0.1 MPa. A high‐pressure hose was providing a connection between generator and chamber while allowing for positioning movement of the chamber. Ultrapure water was used as hydraulic fluid. The maximum system pressure was limited to 200 MPa by the applied high‐pressure hose, however, finite element simulations predicted that the *PiezoGRIN* chamber was not able to withstand pressures that were significantly higher than that. The destructive determination of the chamber burst pressure was not performed to conserve chamber integrity for further experimental sets. However, the most probable correlates of failure might be found in the lens seating or the GRIN lenses themselves. Even though the lenses created a rather thick chamber window, the limited mechanical strength of the BK7 based material itself might cause mechanical failure of the chamber. In case the lenses themselves stayed intact up to that point, a finite element simulation predicted mechanical failure of the lens fitting at about 370 MPa in the current chamber design.

Before first use of the pressure chamber, the PTFE ring separating sample volume and pressure capillary was installed into the chamber by placing the ring and sealing the lid once, as shown in Figure 1 F,G. The PTFE ring was set successfully if 0.1 MPa hydrostatic pressure was applied to the open chamber and no hydraulic fluid leaked into the sample volume.

On the optical excitation side of the chamber, a set of two GT‐LFRL‐200‐023‐50‐CC (810) lenses was used, and on the emission side, a set of two GT‐LFRL‐200‐023‐50‐CC (670) lenses. A set of standard length GRIN lenses was used instead of single custom‐length lenses to take advantage of low mass production prices (25 Euro per lens). The lens fitting bores were drilled with H7 tolerance. After placing the lenses, the gaps were completely filled with Norland Products NOA 81 optical adhesive and cured.

The sequential ray tracing simulations were performed in Zemax Optical Studio 18.1. The simulation setup consisted of a set of GRIN lenses with a pitch of 0.23 each, the lens material property and design files were provided by the lens manufacturer. The lens pair was illuminated by an on axis point light source of 810 nm with the aperture set to an object space NA of 0.5, mimicking the maximum NA of the GRIN lenses. The 1p PSFs in the image plane were generated using the fast Fourier transform Fraunhofer method.^27^ Multiple image plane PSFs were generated along the *z*‐axis by shifting the image plane through the focal spot in increments of 2 µm. The well‐described relation between same excitation wavelength 1p and 2p intensity distributions^28^, ^29^ I_2p_(*x*, *y*, *z*) ∝ I^2^_1p_(*x*, *y*, *z*) was used to estimate 2p PSFs from simulated 1p PSFs.

The microscopy setup that was used to excite the sample with 810 nm 150 fs light pulses at a mean power of 16 mW and a repetition rate of 80 MHz was previously described by Diermeier et al.^30^ with the difference that collecting transmitted signal was performed with a Nikon S Fluor 10×/0.50 objective. The PSF of the setup was determined by imaging orange (620 nm wavelength) fluorescent beads of 100 nm diameter that were embedded in an agarose hydrogel at a pressure of 200 MPa, representing the most demanding application condition in terms of hydrostatic pressure in our system. However, PSF measurements at lower pressures yielded comparable PSF values as the ones at 200 MPa (data not shown). The fluorescence intensity in transmission direction was recorded, and the optical resolution was determined by the FWHM of the resulting PSF. The mean PSF FWHM was determined by analyzing a 3D image stack that contained multiple beads and was spanning a volume of 86 × 86 × 106 µm with the PSFj^31^ software package (Build 243). PSF visual example representations, as used in Figure 3A–C, were generated from an image stack of a random single bead of the evaluation volume.

All animal experiments were in accordance with the animal welfare regulations laid down by the University of Erlangen‐Nürnberg and in agreement with §4 Abs. 1 and 3 of the Tierschutzgesetz (German Animal Welfare Law). The approval number was TS‐6/2016 (to O.F.).

Black six (C57BL/6N) mice were euthanized by Isofluorane anesthesia followed by cervical dislocation. Hind paws were removed and the IO and EDL dissected in a dish with silicone surface containing Ringer's solution. Dissected muscles were transferred into HKS^30^ to electrically silence the muscle fiber membrane (permanent inactivation of Na_v_1.4 channels) and to reduce the extracellular calcium ion concentration.

Single EDL fibers were isolated mechanically in HKS solution, fixed in phosphate‐buffered saline (PBS) with 0.1% glutaraldehyde and stored at 4 °C. For high‐pressure experiments, EDL fibers were positioned inside the PBS filled pressure vessel with the aid of petroleum jelly droplets and pressurized with 0.3 MPa s^−1^ to a maximum of 200 MPa. 3D image stacks were acquired at 200 and 150 MPa as well as at atmospheric pressure before and after pressurization.

Live IO tissue was stored in HKS solution at 4 °C until usage. The sample volume as well as the pressure capillary of the pressure chamber was filled with HKS solution before a segment of IO tissue was sealed into the chamber. Pressure was increased in steps of 10 MPa with 0.3 MPa s^−1^. The sample was observed in real‐time during pressurization using 2P excitation and at each pressure step below 30 MPa, 3D image stacks were recorded. After reaching 30 MPa, a time‐lapse recording was initiated to capture fiber contracture.

## Conflict of Interest

The authors declare no conflict of interest.

## Supporting information

Supplementary

Supplementary

Supplementary
