# Supplementary material for: PiezoGRIN: A High‐Pressure Chamber Incorporating GRIN Lenses for High‐Resolution 3D‐Microscopy of living Cells and Tissues
Source: Adv Sci (Weinh). 2018 Dec 14;6(4):1801453. doi: 10.1002/advs.201801453 (PMC6382305; doi:10.1002/advs.201801453)
Supplement: Supplementary file 1 — Supplementary [file ADVS-6-1801453-s002.pdf]

Copyright WILEY-VCH Verlag GmbH & Co. KGaA, 69469 Weinheim, Germany, 2018.

ADVANCED SCIENCE
Open Access

Supporting Information

for *Adv. Sci.,* DOI: 10.1002/advs.201801453

PiezoGRIN: A High-Pressure Chamber Incorporating GRIN Lenses for High-Resolution 3D-Microscopy of living Cells and Tissues

*Dominik Schneidereit, Sebastian Schürmann, and Oliver Friedrich**

Copyright WILEY-VCH Verlag GmbH & Co. KGaA, 69469 Weinheim, Germany, 2016.

Supporting Information

Video file S1 (S1.avi)
Video file showing contracture of IO muscle fibers.

Vessel blueprints S2 (S2.pdf)
Complete set of blueprints of the chamber vessel.

Dominik Schneidereit,[1,2] Sebastian Schürmann,[1,2] Oliver Friedrich[1,2,3]*
