# Supplementary material for: PiezoGRIN: A High‐Pressure Chamber Incorporating GRIN Lenses for High‐Resolution 3D‐Microscopy of living Cells and Tissues
Source: Adv Sci (Weinh). 2018 Dec 14;6(4):1801453. doi: 10.1002/advs.201801453 (PMC6382305; doi:10.1002/advs.201801453)
Supplement: Supplementary file 2 — Supplementary [file ADVS-6-1801453-s003.pdf]

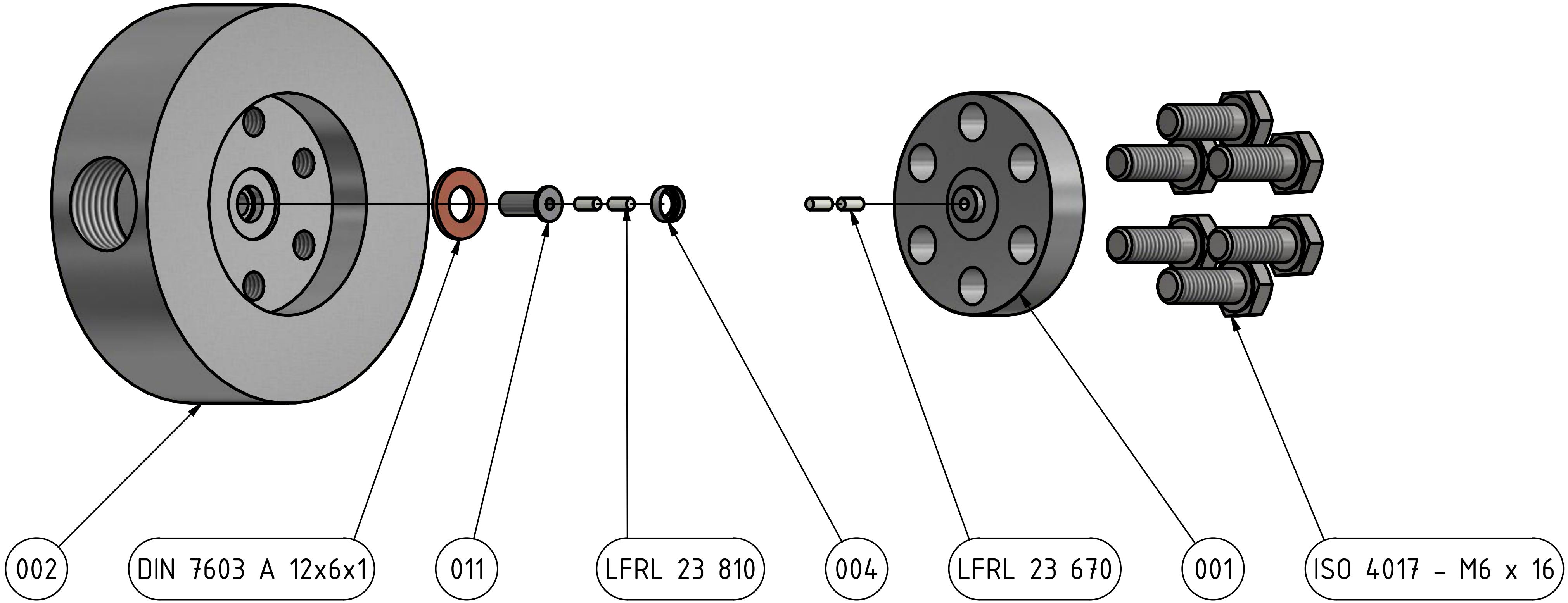

Projekt:

HP-GRIN

DIN ISO 2768 -

DIN ISO 1302

1:1

	Date	Name
Created	18.04.2018	Schneidereit

Overview

Revision 004.3

Institute of Medical Biotechnology

1 /1

A4

State	Changes	Date	Name

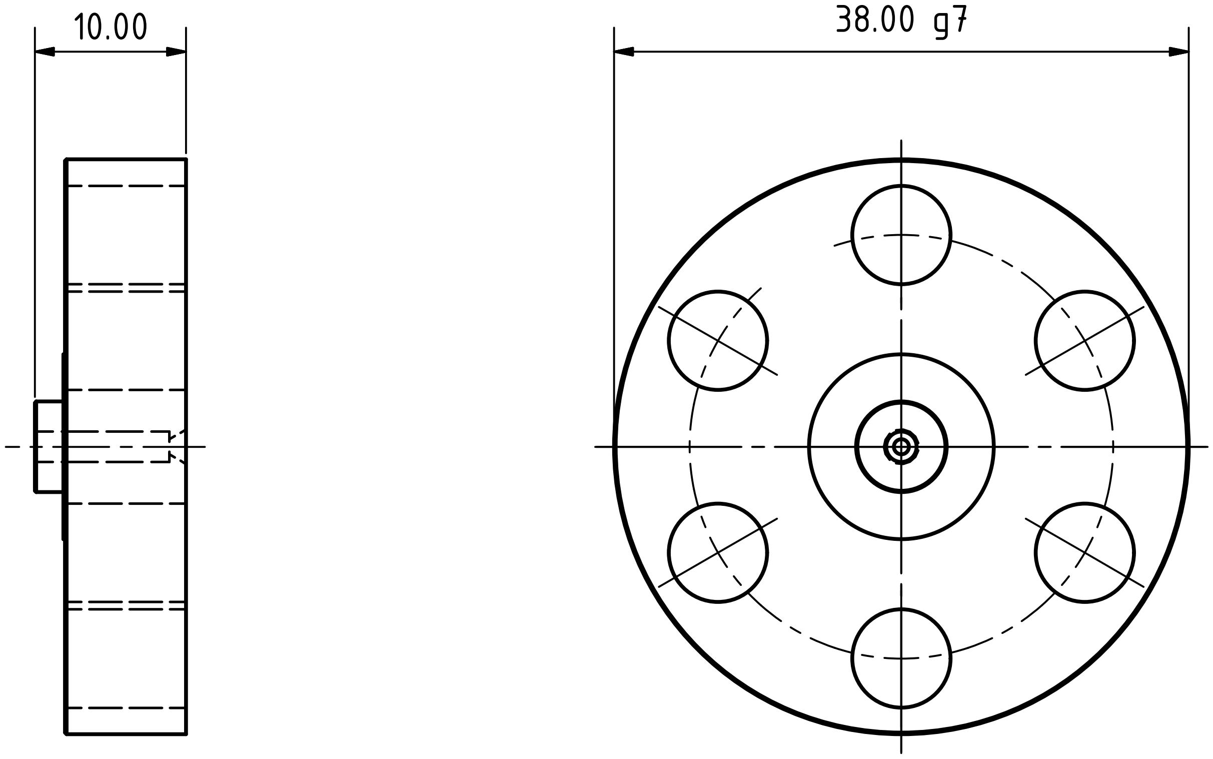

(1 : 1)

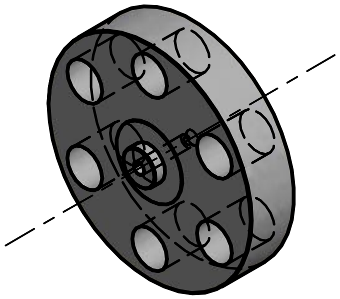

Projekt: HP-GRIN	DIN ISO 2768 - fk	DIN ISO 1302	2:1
			Material Steel 1.4104
	Date	Name	
	Created 18.04.2018	Schneidereit	Chamber Lid<br>Revision 004
	Institute of Medical Biotechnology		Part 001 — 1 /2 — A4

State	Changes	Date	Name

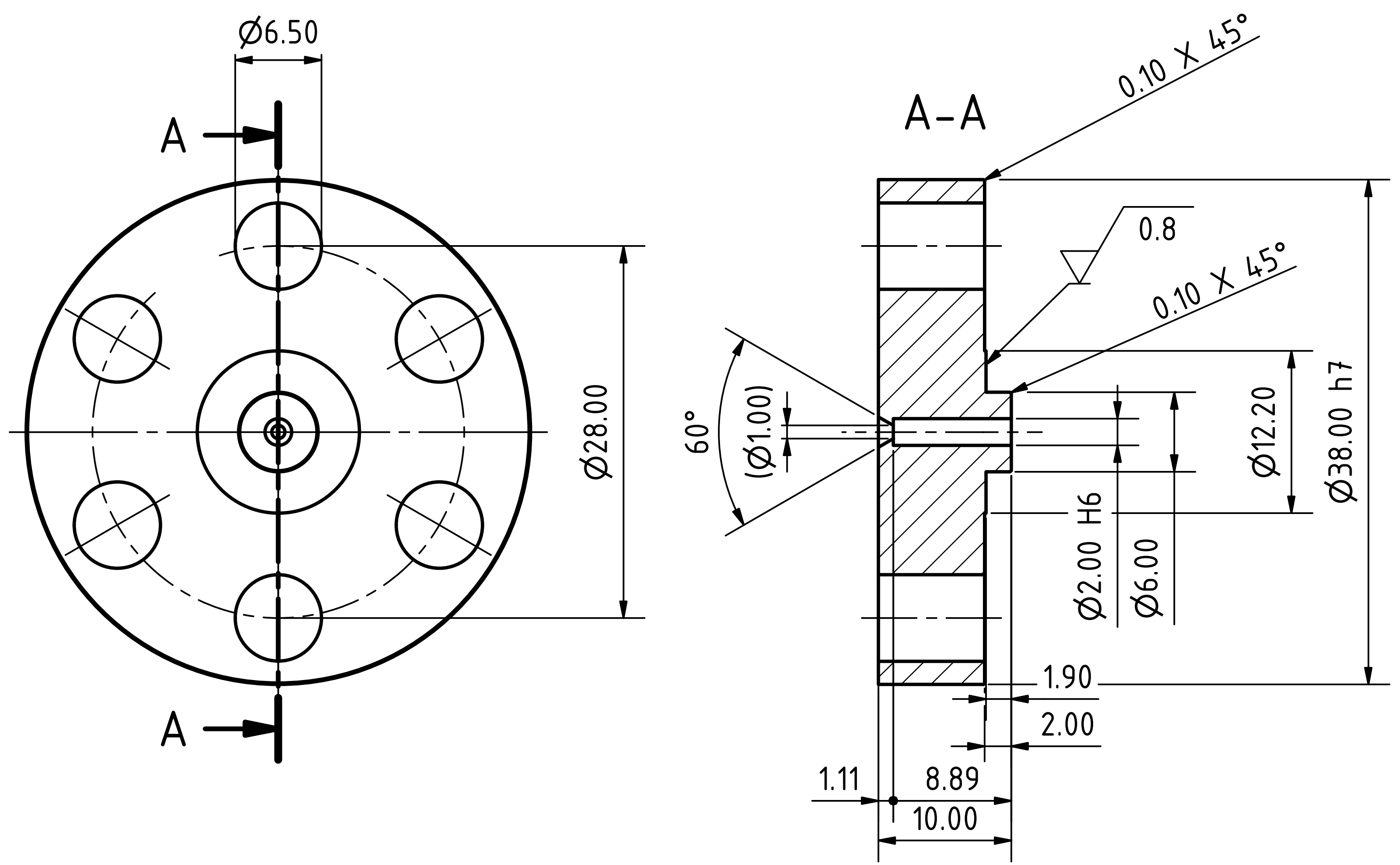

Projekt: HP-GRIN	DIN ISO 2768 - fk	DIN ISO 1302	2:1
			Material Steel 1.4104

State	Changes	Date	Name		Date	Name
				Created	18.04.2018	Schneidereit

Institute of Medical Biotechnology

Chamber Lid

Revision 004

Part 001

2 /2

A4

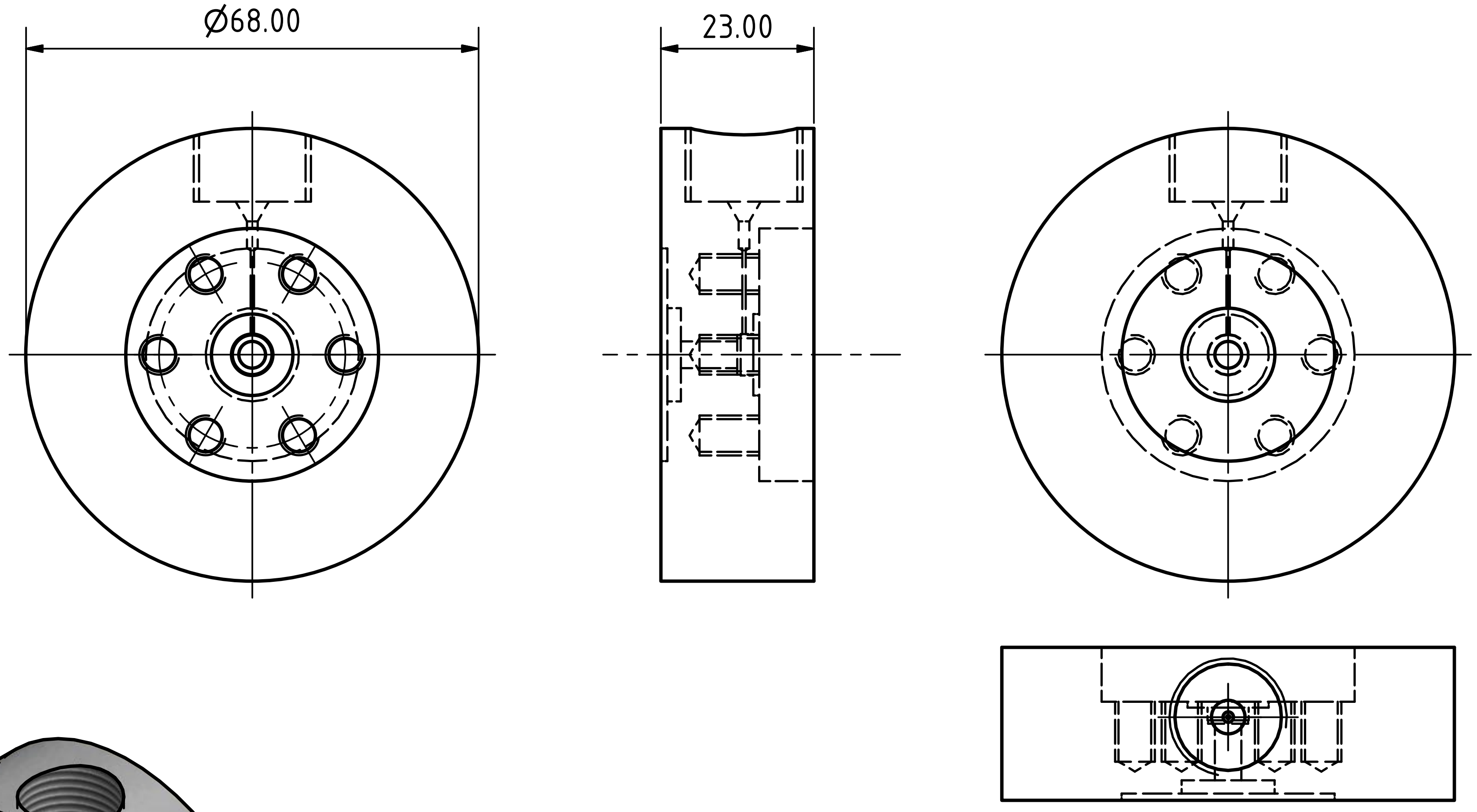

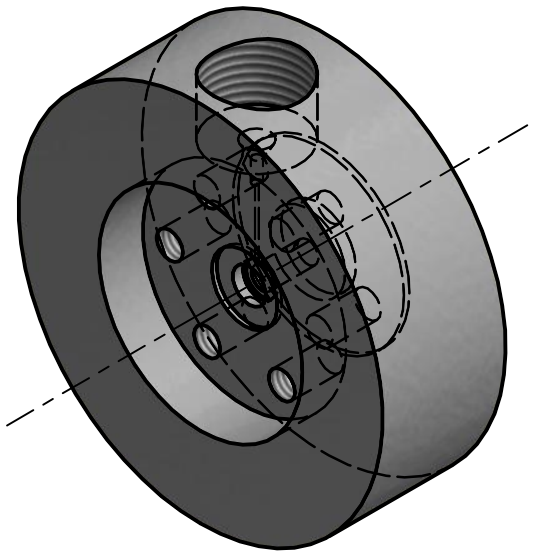

Projekt: HP-GRIN				DIN ISO 2768 - fk	DIN ISO 1302	1:1	
						Material Steel 1.4104	
					Date	Name	Chamber Body<br>Revision 004
				Created	18.04.2018	Schneidereit	
				Institute of Medical Biotechnology			Part 002
							1 /3<br>A4
State	Changes	Date	Name				

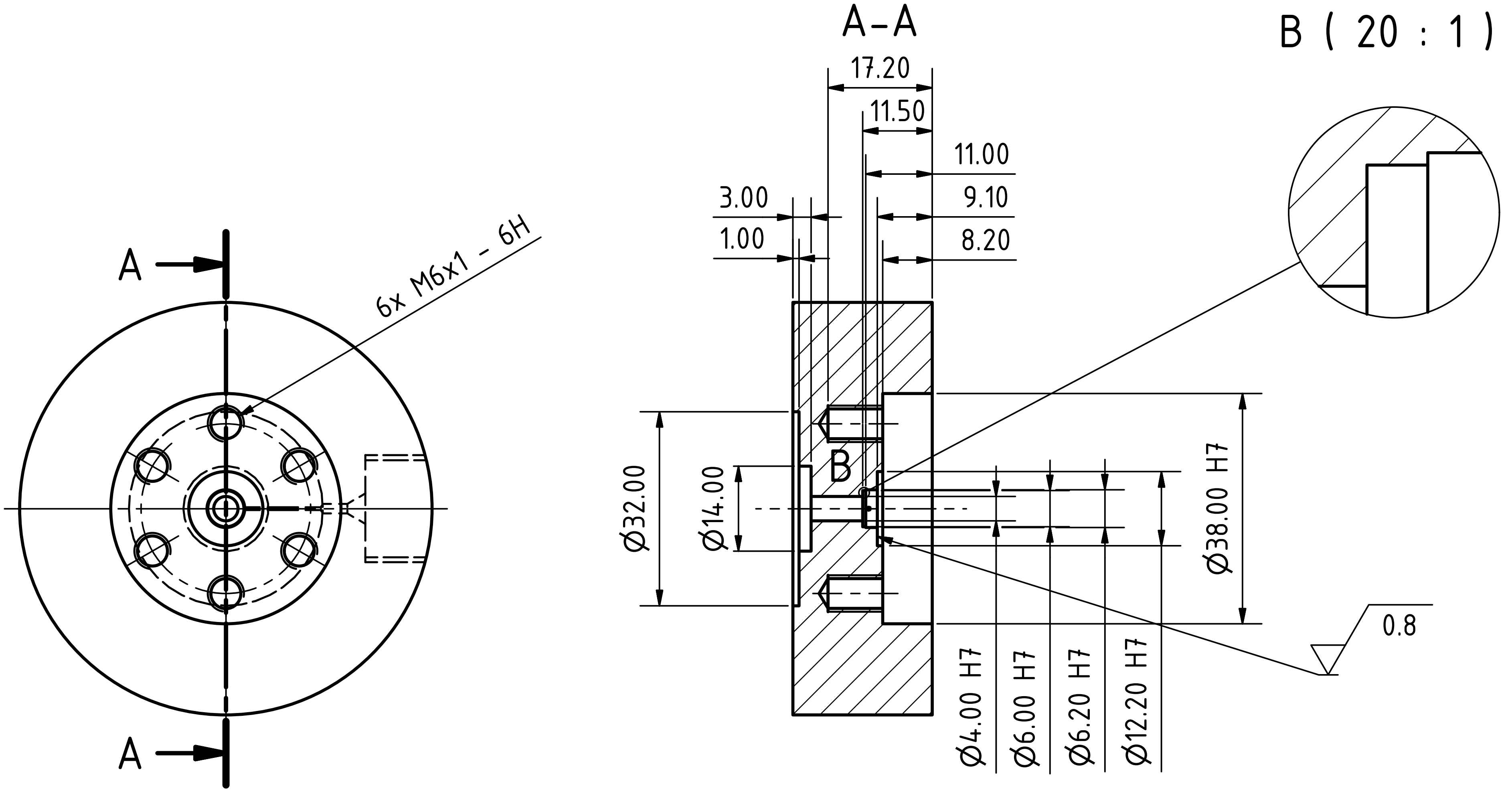

Projekt: HP-GRIN	DIN ISO 2768 - fk	DIN ISO 1302	1:1
			Material Steel 1.4104
	Date	Name	Chamber Body
Created	18.04.2018	Schneidereit	Revision 004
Institute of Medical Biotechnology			Part 002
			2 /3
			A4

State	Changes	Date	Name

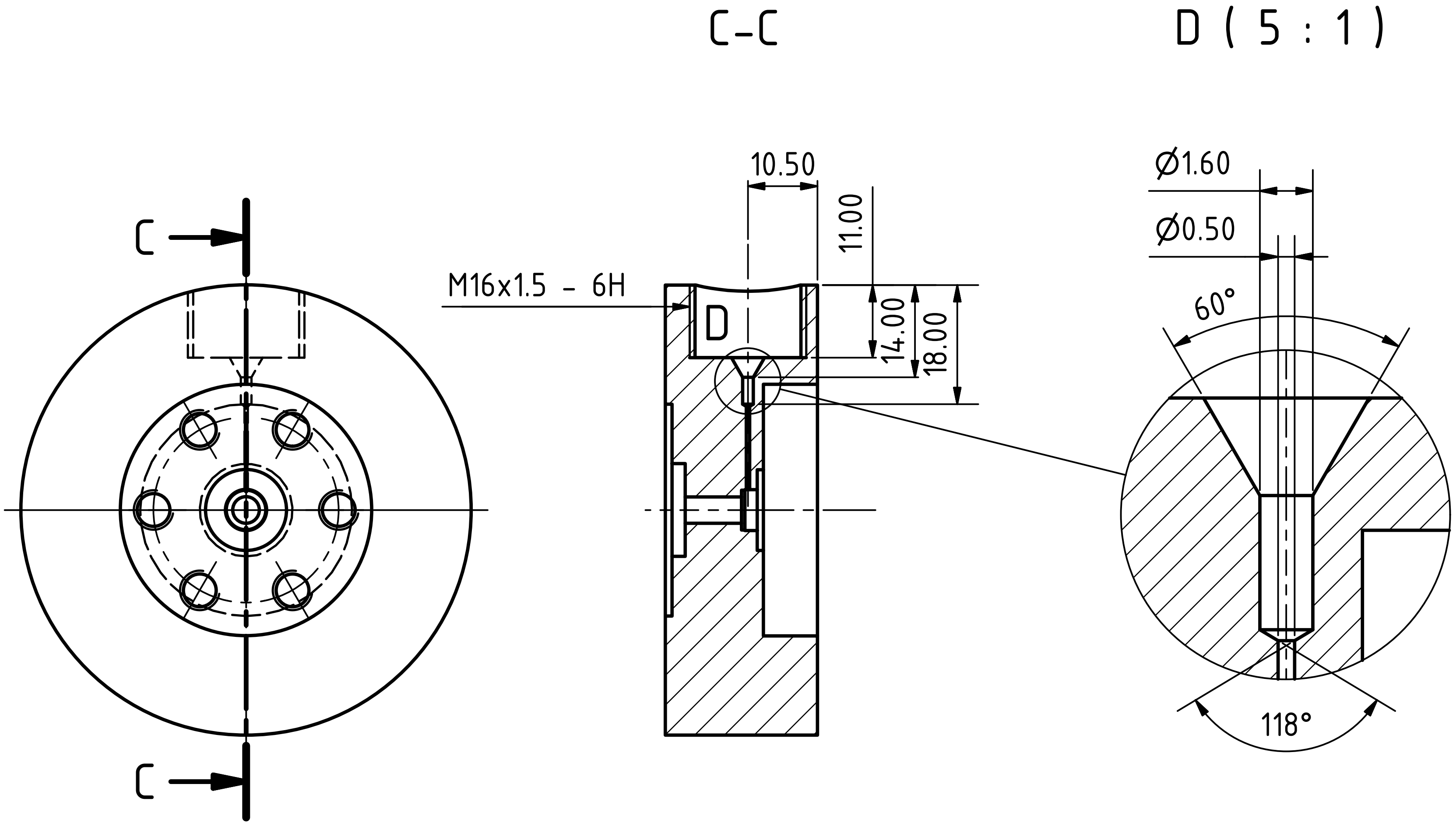

Projekt:
HP-GRIN

DIN ISO 2768 - fk

DIN ISO 1302

1:1

Material Steel 1.4104

	Date	Name
Created	18.04.2018	Schneidereit

Chamber Body
Revision 004

Institute of Medical Biotechnology

Part 002

3 /3
A4

State	Changes	Date	Name

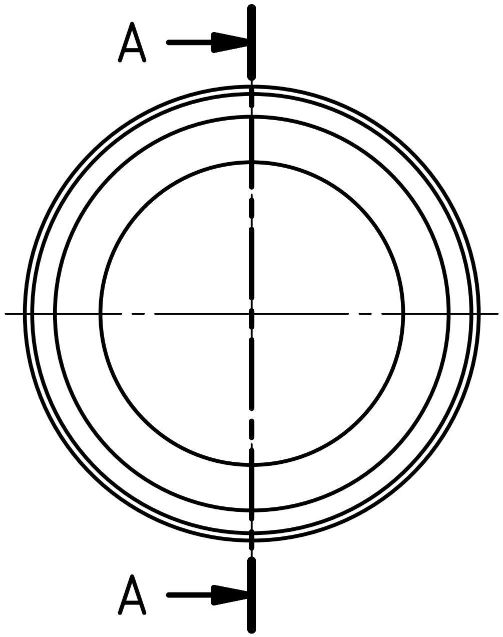

A-A

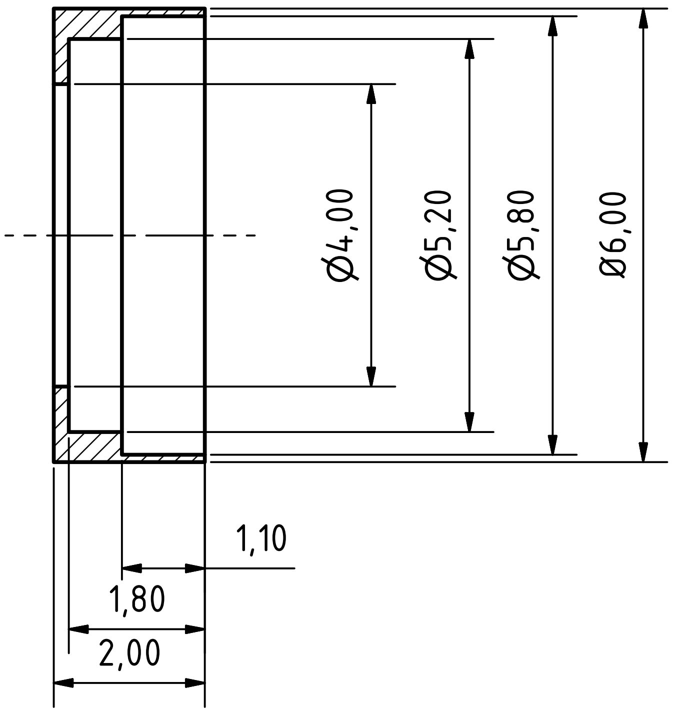

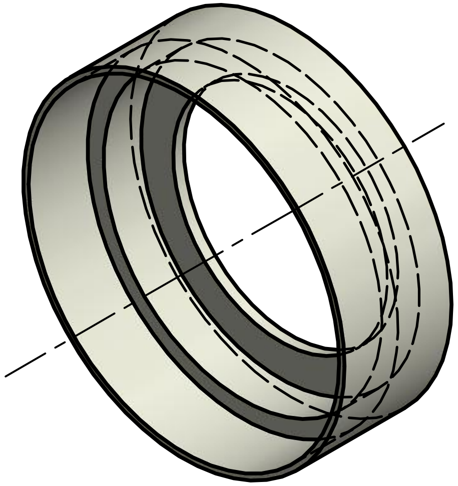

Projekt: HP-GRIN	DIN ISO 2768 - mk	DIN ISO 1302	10:1
			Material PTFE
		Date	Name
	Created	18.04.2018	Schneidereit
			Barrier ring<br>Revision 004
	Institute of Medical Biotechnology		Part 004 — 1 /1 — A4
State / Changes / Date / Name			

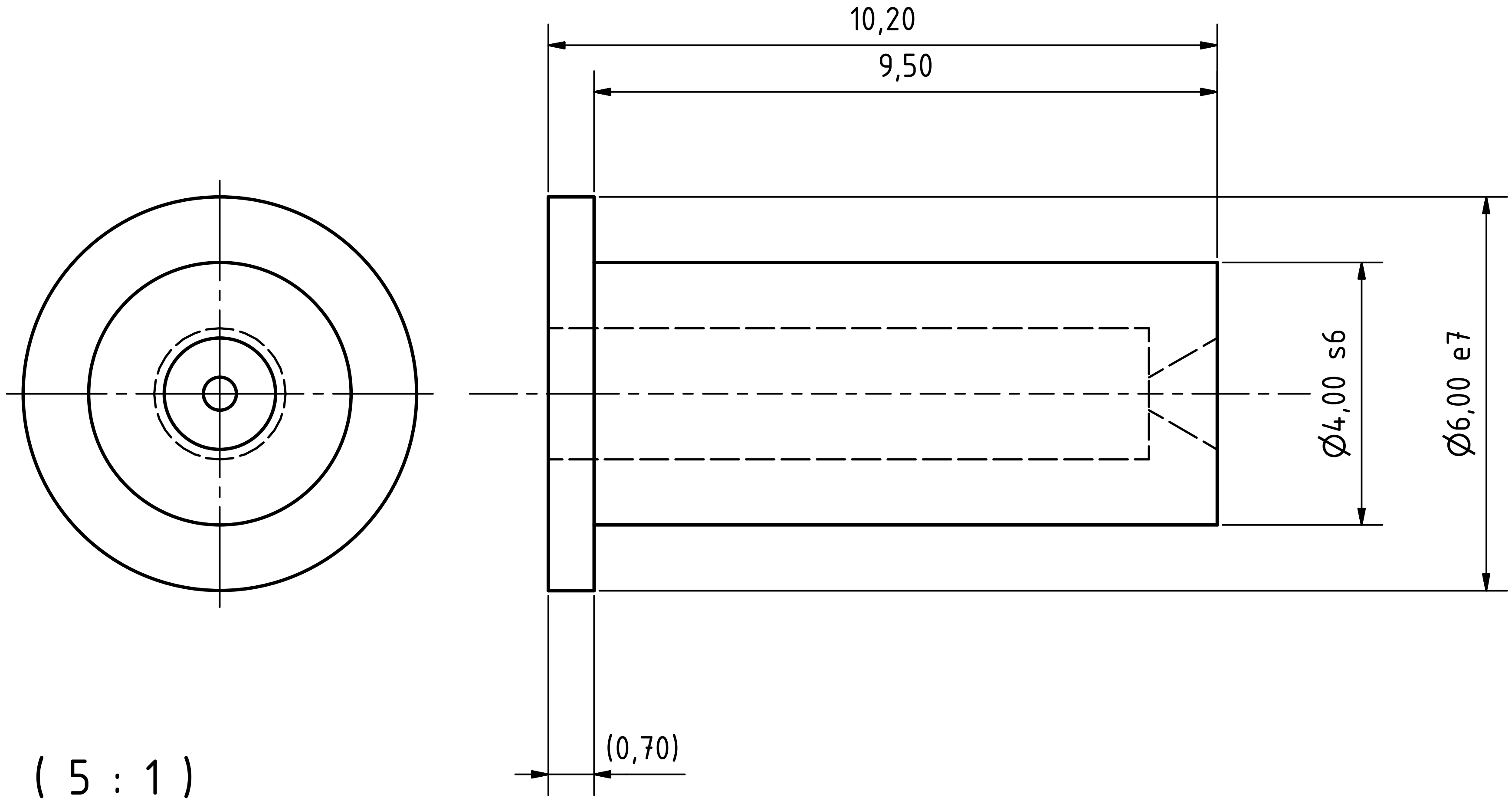

(5 : 1)

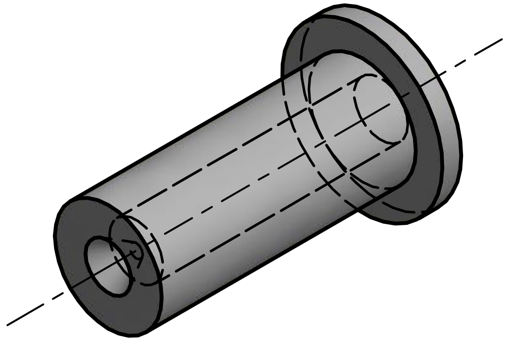

Projekt: HP-GRIN			DIN ISO 2768 - fk	DIN ISO 1302	10:1	
					Material Steel 1.4104	
				Date	Name	Lens bolt
			Created	18.04.2018	Schneidereit	Revision 006
			Institute of Medical Biotechnology		Part 011	1 /2 A4
State	Changes	Date	Name			

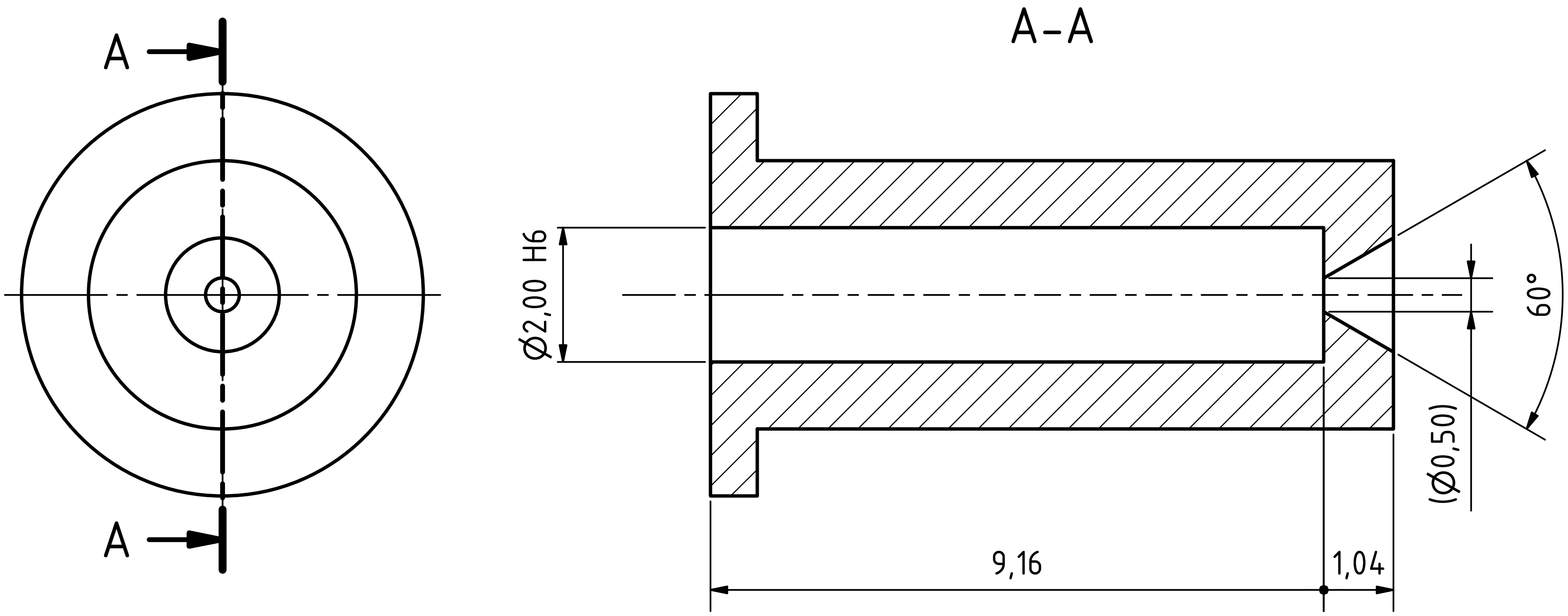

Projekt:
HP-GRIN

DIN ISO 2768 - fk

DIN ISO 1302

10:1

Material Steel 1.4104

	Date	Name
Created	18.04.2018	Schneidereit

Lens bolt

Revision 006

Institute of Medical Biotechnology

Part 011

2 /2

A4

State	Changes	Date	Name
